# Shorebird Use of Coastal Wetland and Barrier Island Habitat in the Gulf of Mexico

**DOI:** 10.1100/tsw.2002.112

**Published:** 2002-02-27

**Authors:** Kim Withers

**Affiliations:** Center for Coastal Studies, Texas A&M University-Corpus Christi, 6300 Ocean Drive, Corpus Christi, Texas 78412, USA

**Keywords:** shorebirds, coastal wetlands, habitat use, Gulf of Mexico

## Abstract

The Gulf Coast contains some of the most important shorebird habitats in North America. This area encompasses a diverse mixture of estuarine and barrier island habitats with varying amounts of freshwater swamps and marshes, bottomland hardwood forests, and coastal prairie that has been largely altered for rice and crawfish production, temporary ponds, and river floodplain habitat. For the purposes of this review, discussion is confined to general patterns of shorebird abundance, distribution, and macro- and microhabitat use in natural coastal, estuarine, and barrier island habitats on the Gulf of Mexico Coast. The following geographic regions are considered: Northwestern Gulf (Rio Grande to Louisiana-Mississippi border), Northeastern Gulf (Mississippi to Florida Keys), and Mexico (Rio Grande to Cabo Catoche [Yucatan Strait]).

Wintering and migrating shorebirds are most abundant along the Gulf Coast in Texas and Tamaulipas, particularly the Laguna Madre ecosystem. Other important areas are the Southwest Coast region of Florida and the area between Laguna Terminos and Puerto Progresso in Mexico. In general, relative abundances of shorebirds increase from north to south, and decrease south of the Tropic of Cancer (23° 27’ N). Based on bimonthly maximum counts within 5° latitudinal bands, the region between 25–30° N is used most heavily by wintering and spring migrating birds.

Non-vegetated coastal wetland habitats associated with bays, inlets and lagoons, particularly tidal flats, and sandy beaches are the habitats that appear to be favored by wintering and migrating shorebirds. In general, these habitats tend to occur as habitat complexes that allow for movement between them in relation to tidal flooding of bay-shore habitats. This relationship is particularly important to Piping Plover and may be important to others.

Although vegetated habitats are used by some species, they do not appear to attract large numbers of birds. This habitat is most widespread between the Texas-Louisiana border and the Florida Panhandle region, but it has not been studied extensively. Shorebird abundance and habitat use in this area need to be addressed.

## INTRODUCTION

The habitat needs of shorebirds (Charadrii), particularly in wintering and migratory staging areas such as the Gulf Coast, were virtually ignored in the U.S. until the Piping Plover (*Charadrius melodus*) was federally listed as endangered/threatened in 1985. Populations of many species of shorebirds are declining due to loss of wetland habitats in breeding, wintering, and migratory staging areas[1,2]. Wintering and migrating shorebirds spend the majority of their time feeding, and most shorebird mortality in winter has been related either directly or indirectly to food shortages[3]. Many shorebirds exhibit site fidelity[4,5], and tend to concentrate in a few sites that are often separated by great distances and where food resources are relatively abundant. Thus, shorebirds are particularly vulnerable to population declines due to foraging habitat loss in nonbreeding areas like the Gulf Coast.

Shorebird breeding ranges in the Western Hemisphere generally begin near 30º N and extend north of the Arctic Circle. Wintering ranges extend from about 40º N to 55º S (Fig. 1, Table 1) with a few species staying as far north as 60°N. Several species remain within some areas year-round (Tables 1 and 2). The majority of shorebirds breeding from 30–50º N is found in the Great Plains and Great Basin of North America. Those that breed in higher latitudes are generally found along coastlines, including the shore of Hudson Bay, although the interior is used by some species breeding between 50º and 70º N. The distance that shorebirds migrate is classified as long (>14,000 km), intermediate (6,000–12,000 km), or short (>5000 km)[6].

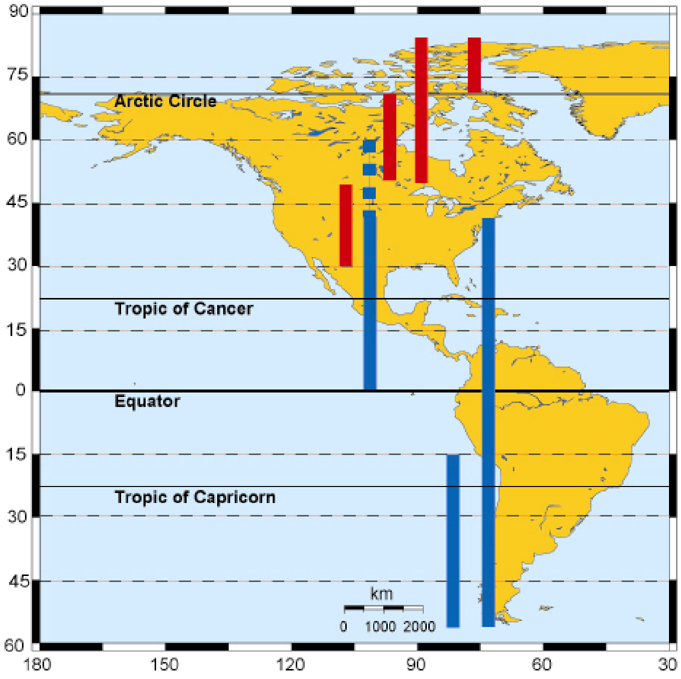

**FIGURE 1.** The Western Hemisphere showing shorebird breeding (red bars) and wintering (blue bars) ranges. See Table 1 for additional information.

## TABLE 1
## Geographic Matrix of Shorebird Breeding and Wintering Ranges[a]

| WINTER RANGE | BREEDING RANGE: 30–50° N | 50–70° N | Trans-Arctic Circle (>50°N) | Above Arctic Circle (>70°N) |
|---|---|---|---|---|
| North of Equator<br>Equator north to 40-60° N | American Avocet*<br>US<br>Long-billed Curlew<br>US<br>Marbled Godwit<br>WS<br>Mountain Plover<br>US<br>Piping Plover<br>NS<br>Killdeer*<br>WS<br>Snowy Plover*<br>NS | Long-billed Dowitcher<br>WI | Dunlin<br>JI | |
| Trans-equatorial<br>40° N to 55° S | Black-necked Stilt*<br>NS<br>Spotted Sandpiper<br>WI<br>Willet*<br>WS<br>Common Snipe*<br>US<br>American Oystercatcher*<br>UI (breeds Atlantic coast only) | Short-billed Dowitcher<br>N/WI<br>Greater Yellowlegs<br>NI<br>Least Sandpiper<br>N/WI<br>Western Sandpiper<br>CI<br>Whimbrel<br>WI | Black-bellied Plover<br>WI<br>Lesser Yellowlegs<br>NI<br>Ruddy Turnstone<br>JI<br>Semipalmated Plover<br>NI<br>Semipalmated Sandpiper<br>NI | Sanderling<br>JI<br>Red Knot<br>JI<br>Solitary Sandpiper<br>WI<br>Stilt Sandpiper<br>NL |
| South of 15° S only | Wilson's Phalarope<br>WI | Hudsonian Godwit<br>NL | American Golden Plover<br>NL<br>Baird's Sandpiper<br>NL<br>Pectoral Sandpiper<br>NL | Buff-breasted Sandpiper<br>NL<br>White-rumped Sandpiper<br>NL |

[a]Migration pattern and distance (see text) codes follow species common name (compiled from [6]).
US = unspecified pattern, short distance; UI = unspecified pattern, intermediate distance; WS = widespread pattern, short distance; WI = widespread pattern, intermediate distance; JI = jump pattern, intermediate distance; NS = narrow pattern, short distance; NI = narrow pattern, intermediate distance; NL = narrow pattern, long distance; N/WI = narrow or widespread pattern, intermediate distance; CI = crossband pattern, intermediate distance.
*Species with year-round ranges, see Table 2.

## TABLE 2
## Year-Round Ranges and Breeding and/or Wintering Ranges for Species that Could Not Be Categorized in Table 1

| Species | Range |
|---|---|
| American Avocet | **Year Round:** 18-40° N |
| Black-Necked Stilt | **Breeding:** 30-40° N, western US only<br>**Year Round:** 30° N to 40° S<br>**Wintering:** unspecified |
| Common Snipe | **Year Round:** 30-40° N, Great Basin only |
| Killdeer | **Year Round:** 20-35° N; 5-15° S |
| Snowy Plover | **Year Round:** Pacific coast – 30-50° N, 5-40° S; Gulf & Caribbean coast 5-30° N |
| Willet | **Year Round:** Atlantic, Gulf & Caribbean coasts 20-40° N |
| Wilson's Plover | **Breeding:** Gulf Coast through mid-Atlantic coast<br>**Year Round:** Atlantic, Gulf, & Caribbean coasts 20-30° N<br>**Wintering:** Pacific & Caribbean coasts 20° N to 10° S |
| American Oystercatcher | **Year Round:** Gulf of Mexico & Pacific coasts 20-30° N, Atlantic coast 30-40° N<br>**Wintering:** Atlantic and Gulf Coast of Florida ~27-31° N only |
| American Woodcock | **Breeding:** 35-50° N, east of Great Plains only<br>**Wintering:** 25-45° N, east of Texas/Oklahoma Panhandle only |

Most of the birds breeding between 30–50º N are short-distance migrants, and the majority that winter south of 15º S is long-distance migrants (Table 1). Five migration patterns have been determined in midcontinental U.S. populations (Fig. 2)[6]. Birds such as Ruddy Turnstone (*Arenaria interpres*) leave the breeding area and arrive on the wintering grounds with few or no stops in between, jumping from one area to the other. Only four species exhibit this pattern (Table 1), and all are intermediate-distance migrants. Birds that migrate in a narrow band (Fig. 2) are concentrated in a small area east to west, but are dispersed from north to south. All long-distance migrants, five intermediate-distance migrants, and one short-distance migrant exhibit the narrow-band pattern (Table 1). With the exception of Black-Necked Stilt (*Himantopus mexicanus*), these birds breed above 50º N. The widespread pattern of migration (Fig. 2) is exhibited by seven species, and all but Killdeer (*Charadrius vociferus*) are intermediate-distance migrants (Table 1). These birds are dispersed throughout the area with time and latitude playing the major role in determining areas of concentration in any portion of the route north or south. Two species, Short-Billed Dowitcher (*Limnodromous griseus*) and Least Sandpiper (*Calidris minutilla*), migrate in narrow-band and/or widespread pattern. The majority of birds (80–90%) is found within a narrow band, but the remainder are widely dispersed. These two species are generally classified as intermediate-distance migrants, but some individuals may be long-distance migrants. The cross-band pattern (Fig. 2) is exhibited only by Western Sandpipers (*Calidris mauri*). This species is an intermediate-distance migrant that is confined to a relatively narrow east-west corridor, and jumps between areas of concentration.

The Gulf Coast contains some of the most important shorebird habitat in North America. For migrating and wintering shorebirds using the Interior Flyway, the wetlands and barrier islands of this region represent the first large expanses of suitable habitat between northern breeding grounds and more distant wintering grounds in South America. There are also important breeding areas within this region for a few of shorebirds, such as Willet (*Catatrophorus semipalmatus*), American Oystercatcher (*Haematopus palliatus*), and Snowy Plover (*Charadrius alexandrinus*). This area encompasses a diverse mixture of estuarine and barrier island habitat with varying amounts of freshwater swamps and marshes, bottomland hardwood forests, coastal prairie that has been largely altered for rice and crawfish production, temporary ponds, and river floodplain habitat.

Thirty-nine species of shorebirds have been documented along the Gulf of Mexico (Table 3). Of these, the populations of eight species may be declining based on global and North American population estimates[7]: Eskimo Curlew (*Numenius borealis*), Snowy Plover, Piping Plover, Mountain Plover (*Charadrius montanus*), Long-Billed Curlew (*Numenius americanus*), Solitary Sandpiper (*Tringa solitaria*), Buff-Breasted Sandpiper (*Tryngites subruficollis*), and Wilson's Plover (*Charadrius wilsonia*). Eskimo Curlew may be extinct. Piping Plover, Snowy Plover and Long-Billed Curlew are short-distance migrants that breed within the North American Great Plains and Great Basin, winter north of the equator[6] (Table 1), and are subject to habitat loss in all portions of their ranges. Solitary Sandpiper, Buff-Breasted Sandpiper, and Wilson's Plover are classified as "difficult-to-count species"[7], suggesting population estimates may be too low. Solitary Sandpiper winters throughout the wide trans-equatorial band (Table 1). Buff-Breasted Sandpiper winters in the far south. Both breed above the Arctic Circle. Wilson's Plover is found year-round on Atlantic, Gulf, and Caribbean Coasts, with wintering birds in coastal California and the northern Caribbean (Table 2).

## STUDY AREA AND METHODS

For the purposes of this review, discussion is restricted to general patterns of shorebird abundance, distribution, and macro- and microhabitat use in natural coastal, estuarine, and barrier

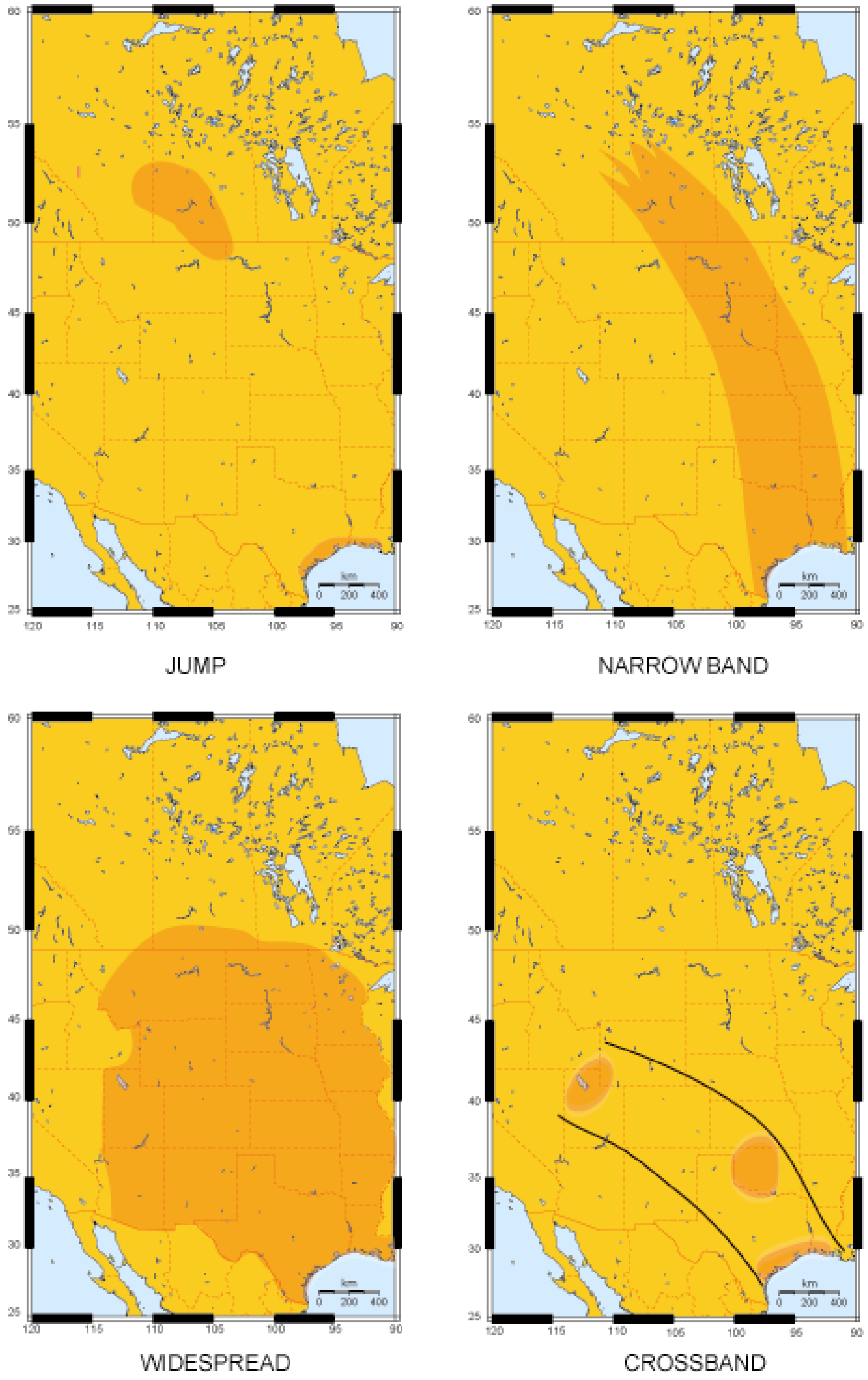

**FIGURE 2.** Migration patterns of shorebirds in midcontinental North America[6].

island habitats on the Gulf of Mexico Coast. It is outside the scope of this paper to review shorebird use of created or restored habitats (including dredged material), agricultural habitats, or inland freshwater wetlands, or to give a detailed overview of each species. The following geographic regions are considered (Fig. 3): Northwestern Gulf (Rio Grande to Louisiana-Mississippi border), Northeastern Gulf (Mississippi to Florida Keys), and Mexico (Rio Grande to Cabo Catoche [Yucatan Strait]).

**TABLE 3**
**Shorebird Seasonal Occurrence, Abundance, and Status and Area Regional Importance to Each Species in the Gulf Region of the U.S.**

| Species | | Season/ Abundance | Regional Importance | | Status | |
|---|---|---|---|---|---|---|
| | | | NW | NE | NW | NE |
| Black-bellied Plover | *Pluvialis squatorola* | M, W, r, s | I | I | HC | M |
| American Golden-Plover | *Pluvialis dominica* | M | V | | HI | M |
| Snowy Plover | *Charadrius alexandrinus* | M, W, b, s | I | I | HC | EH |
| Wilson's Plover | *Charadrius wilsonia* | m, w(tr), B | V | I | HI | H |
| Semipalmated Plover | *Charadrius semipalmatus* | M, w | I | V | | |
| Piping Plover | *Charadrius melodus* | M, W, s | V | I | HI | EH |
| Killdeer | *Charadrius vociferus* | m, w, b | V | I | HC | |
| Mountain Plover | *Charadrius montanus* | m, w(tr) | | * | HC | * |
| American Oystercatcher | *Haematopus palliatus* | W, b | | V | | EH |
| Black-necked Stilt | *Himantopus mexicanus* | M, W, b | I | I | | |
| American Avocet | *Recurvirostra americana* | M, W, b | I | | | M |
| Greater Yellowlegs | *Tringa melanoleuca* | M, W | V | V | | M |
| Lesser Yellowlegs | *Tringa flavipes* | M, W | V | V | | |
| Solitary Sandpiper | *Tringa solitaria* | M, W(tr) | I | V | | H |
| Willet | *Cataptrophorus semipalmatus* | M, W, B, r, s | I | V | | M |
| Spotted Sandpiper | *Actitus macularia* | M, w | I | V | | |
| Upland Sandpiper | *Bartramia longicauda* | M | V | | HI | H |
| Eskimo Curlew | *Numenius borealis* | ? | V | * | HC | * |
| Whimbrel | *Numenius phaeopus* | M | V | V | HI | H |
| Long-billed Curlew | *Numenius americanus* | M, W | V | | HC | H |
| Hudsonian Godwit | *Limosa haemastica* | M | V | | HC | M |
| Marbled Godwit | *Limosa fedoa* | M, W | I | | HC | H |
| Ruddy Turnstone | *Arenaria interpres* | M, W, R | I | I | HC | M |
| Red Knot | *Calidris canutus* | m, w | | V | HC | EH |
| Sanderling | *Calidris alba* | M, W, s | I | V | | M |
| Semipalmated Sandpiper | *Calidris pusilla* | M | I | V | | H |
| Western Sandpiper | *Calidris mauri* | M, W, R | I | I | | M |
| Least Sandpiper | *Calidris minutilla* | M, W | I | I | | M |
| White-rumped Sandpiper | *Calidris fuscicollis* | M | V | | | |
| Baird's Sandpiper | *Calidris bairdii* | m | I | | | |
| Pectoral Sandpiper | *Calidris melanotos* | M | V | V | | M |
| Dunlin | *Calidris alpina* | M, W | I | I | | M |
| Stilt Sandpiper | *Calidris himantopus* | M, w | V | I | HC | H |
| Buff-breasted Sandpiper | *Tryngites subruficollis* | M | I | | | H |
| Short-billed Dowitcher | *Limnodromous griseus* | M, W | I | V | | H |
| Long-billed Dowitcher | *Limnodromous scolopaceus* | M, W | I | | | |
| Common Snipe | *Gallinago gallinago* | w | I | V | HC | M |
| American Woodcock | *Scolopax minor* | w | I | V | HC | H |
| Wilson's Phalarope | *Phalaropus tricolor* | m | | | | M |

Data compiled from [8] and [48].
Season/Abundance: M = common or abundant, fall or spring migration; m = common to uncommon or rare, fall or spring migration; W = common or abundant, wintering; w = common to uncommon or rare, wintering; B = common breeding season; b = small numbers, breeding season; r = small resident populations; s = small numbers of non-breeding birds present during summer; tr = transient. Regional Importance (Northwestern Gulf [NW]; Northeastern Gulf [NE]: V = area of extremely high importance relative to other regions; I = area of considerable importance. Status: Northwestern Gulf (NW); HI = highly imperiled; HC = of high concern; Northeastern Gulf (NE); EH = extremely high priority; H = high priority; M = moderate priority.
*Absent from all or most of the area.

Abundance data were compiled and synthesized from four sources. In the Northwestern Gulf, two datasets were available[6,8]. The data compiled by[6] were collected from multiple sites within midcontinental North America, including Texas and Louisiana, over multiple months, multiple years, and by multiple observers. Although not stated explicitly, it appears that data were collected on the ground. Much of the data for Texas were collected during repeated, systematic surveys of U.S. Fish and Wildlife Service National Wildlife Refuges between 1992 and 1996.

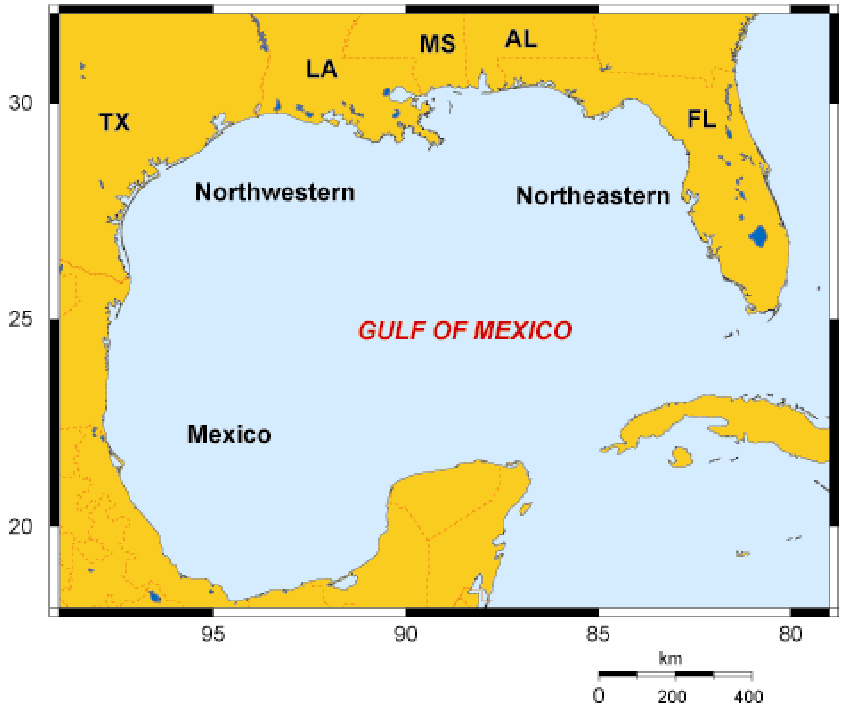

**FIGURE 3.** Geographic regions of the Gulf of Mexico for which shorebird abundance, distribution, and habitat use will be summarized.

Data were presented as maximum counts January–June and July–December. The data presented[8] were collected during aerial surveys of the Texas coast during April 1997 and April 1998. These data represent the total number of birds counted at each site during one survey. For the northeastern Gulf, one dataset was available[9]. In this study, 58 sites along the Florida Gulf coast were surveyed at least four times between December 1993 and March 1994. Sites were visited within one hour of the predicted high and low tides, and shorebirds were counted and identified to species. Data were presented as mean numbers of birds with standard deviation. In Mexico, one dataset was available[10]. Aerial surveys were conducted in 61 zones or locations along the Gulf of Mexico during January 1993. Numbers of shorebirds were determined by counting when flocks were small and by estimation when large concentrations were found.

To illustrate general patterns of shorebird abundance in the northwestern Gulf, I used the maximum count presented by either Skagen et al.[6] or Elliott and McKnight[8] to obtain the best geographic coverage. Relative abundances for Texas were calculated using the 1998 data in Elliott and McKnight's study[8], which had the widest geographic coverage. For Florida, the mean and maximum values (mean + 1 SD) are given to facilitate comparisons among regions. The number of locations in Mexico was reduced by combining counts from several adjacent sites (e.g., Laguna Madre beach, Laguna Madre east side, Laguna Madre west side, Laguna Madre/N of Tampico).

## NORTHWESTERN GULF

Thirty-five of the 39 species are regularly found in substantial numbers in coastal habitats of the Northwestern Gulf[8]. The area provides migration and wintering habitats for over 1 million shorebirds each year, and it is considered highly important to most species relative to other areas in the U.S. and Gulf of Mexico. The entire critical wintering habitat for Piping Plover is found in this area. Piping Plover, Snowy Plover, Eskimo Curlew, and Long-Billed Curlew are considered "highly imperiled" in the Northwestern Gulf. Piping Plovers appear

**TABLE 4**
**Maximum Numbers of Shorebirds in Long, Intermediate, and Short Distance Migrant Categories Counted at Selected Sites in the Northwestern Gulf of Mexico (Louisiana and Texas) January-June and July-December**

| | Long-Distance Migrants | | Intermediate Distance Migrants | | Short Distance Migrants | |
|---|---|---|---|---|---|---|
| | Jan-Jun | Jul-Dec | Jan-Jun | Jul-Dec | Jan-Jun | Jul-Dec |
| Grand Terre Island | | | 31,740 | | | |
| Anahuac NWR | | | 18,557 | | | |
| Bolivar Flats | | | 35,244 | | 17,664 | 4,778 |
| Brazoria NWR | | 2,519 | 38,479 | | 2,465 | 4,766 |
| San Bernard NWR | | | 29,892 | | 2,437 | |
| Matagorda NWR | | | 16,143 | | 4,874 | |
| Tule Lake, Corpus Christi | | | | | 2,001 | |
| Western Nueces Co. | | | | | 1,972 | |
| Laguna Atascosa | 9,170 | | 70,532 | 21,140 | 5,350 | 3,722 |
| South Padre Island | | | 14,876 | | | |
| Boca Chica Beach | | | | | 6,173 | |

Data from [6].

to be most abundant in Texas[6]. This bird was present 25–30° in all months except June and is most abundant in this latitudinal range. Overall abundance in the region peaked at over 500 individuals during mid- to late fall (August–October) and mid-winter through spring migration (January–early April). Snowy Plover are most abundant in the breeding areas of the Great Plains and Great Basin, but were present throughout the year and breed along the Texas Gulf Coast. Peaks in abundance occurred during fall and spring migration and winter. The six highest counts of Long-Billed Curlew were in Texas, but in more inland coastal habitats such as agricultural land. Overall, the population of this species present 25–30° N is low. However, large peaks occur in late summer and mid-winter, and these peaks represent the largest concentrations (~2,000 individuals) that occur at any time or place throughout its range. In general, intermediate- and short-distance migrants are the most abundant shorebirds in the region with largest concentrations occurring January–June (Table 4).

## Regional Habitat Use and Abundance

The area between the Rio Grande in Texas and the Louisiana-Mississippi border contains a diverse mixture of coastal and estuarine wetlands[8]: Mississippi River Coastal Wetlands, Chenier Plain, Texas Mid-Coast, and Laguna Madre (Fig. 4). The Mississippi River Coastal Wetlands contain coastal marsh, deltaic flats and tidal marshes, barrier islands, mud flats, and estuarine bays. The estuarine portions of the Chenier Plain and Texas Mid-Coast are dominated by coastal marsh, including some tidal flat areas, particularly in Texas. The Laguna Madre system encompasses both the Laguna Madre in Texas and Laguna Madre de Tamaulipas in Mexico. This system is dominated by huge expanses of tidal flats bordering the hypersaline lagoons, barrier islands, and a large wetland complex in the Rio Grande Delta. There is little quantitative information concerning shorebird abundance or habitat use in this region with the exception of habitats within Texas.

Much of the coastal habitat within the Mississippi River Coastal Wetlands is relatively inaccessible and may hold large concentrations of shorebirds[8], but their distribution and abundance are not well known. A maximum of nearly 35,000 individuals was counted on Grand Terre Island (GTI) tidal wetlands and barrier island beaches January–June (Fig. 5)[6]. Other areas identified as important to shorebirds in this region are tidal mudflats and deltaic splays at Delta National Wildlife Refuge, Pass-a-Loutre Wildlife Management Area, Grand Isle and Chandeleur Islands beaches and wetlands, tidal wetlands and mudflats of Atchafalaya Bay, and the brackish ponds and wetlands of the Bonnet Carre Spillway[8].

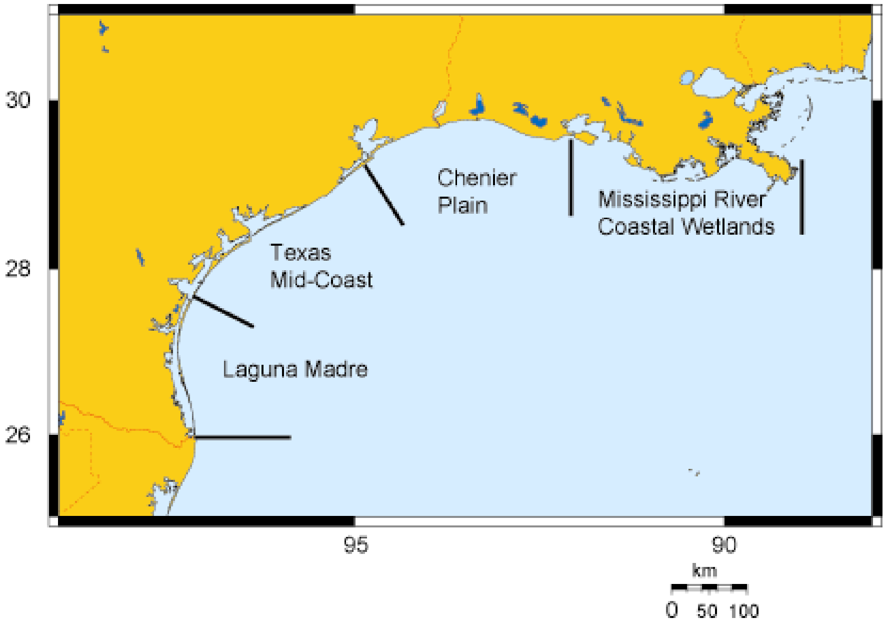

**FIGURE 4.** Regional habitat types within the Northwestern Gulf of Mexico[8].

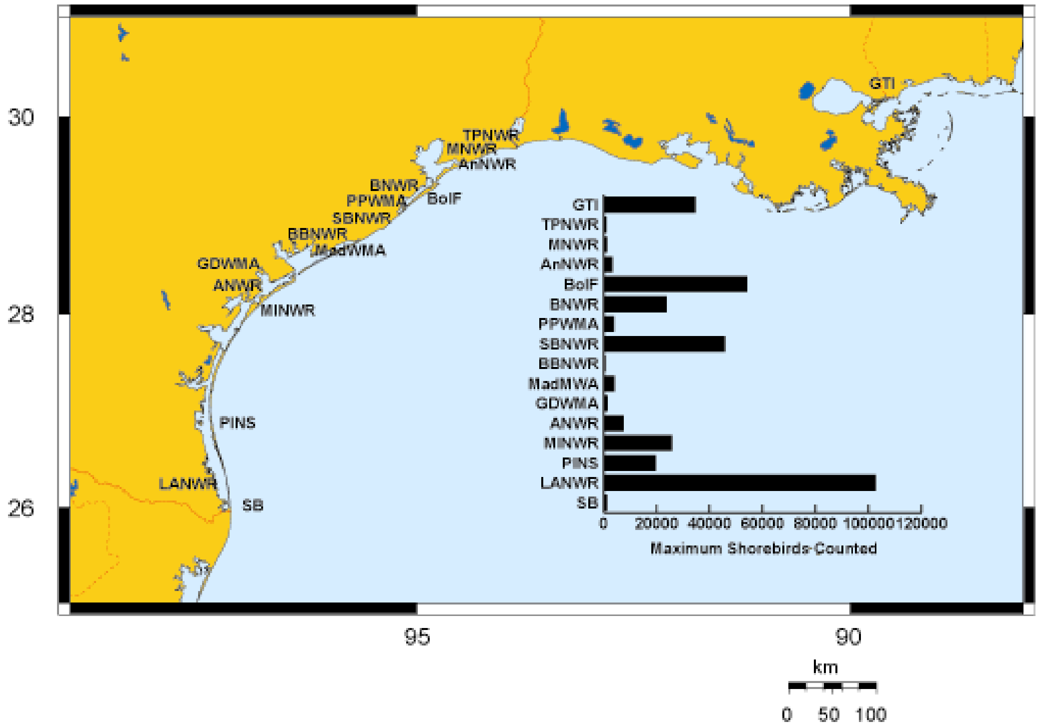

**FIGURE 5.** Shorebird abundance and distribution in the Northwestern Gulf presented as maximum[6] or total counts[8]. GTI = Grand Terre Island, Jefferson Parish, Louisiana[6]; TPNWR = Texas Point National Wildlife Refuge[8]; MNWR = MacFaddin National Wildlife Refuge[8]; AnNWR = Anahuac National Wildlife Refuge[8]; BolF = Bolivar Flats[6]; BNWR = Brazoria National Wildlife Refuge[6]; PPWMA = Peach Point Wildlife Management Area[8]; SBNWR = San Bernard National Wildlife Refuge[6]; BBNWR = Big Boggy National Wildlife Refuge[8]; MadWMA = Mad Island Wildlife Management Area[8]; GDWMA = Guadalupe Delta Wildlife Management Area[8]; ANWR = Aransas National Wildlife Refuge[8]; MINWR = Matagorda Island National Wildlife Refuge[6]; PINS = Padre Island National Seashore[8]; LANWR = Laguna Atascosa National Wildlife Refuge[6]; SB = South Bay[8].

In the Chenier Plain, much of the shorebird habitat along the coast consists of brackish or saline marsh. Important shorebird sites include the East Cameron Jetties at the mouth of the Calcasieu River, Sabine National Wildlife Refuge, Lacassine National Wildlife Refuge, Anahuac National Wildlife Refuge (AnNWR), and Bolivar Flats (BolF)[8]. The beaches and bay-shore habitats of the Bolivar Peninsula on the upper Texas Coast are used by large numbers of shorebirds with a maximum count exceeding 50,000 January–June (Fig. 5) and of over 13,000 July–December[6]. Bolivar Flats has been recognized by the Western Hemisphere Shorebird Reserve Network (WHRSN) as a site of “international significance,” an area that hosts at least 100,000 shorebirds annually or at least 10 percent of the species flyway population based on peak counts. It was the site with the tenth highest count in surveys of mid-continental shorebird populations[6]. This area is of particular importance to Piping Plover with maximum counts from 200–300[6] and has been studied extensively in that regard[11,12,13,14,15,16,17,18].

Coastal marsh is present only in a narrow band at the Gulf/land interface in the Texas Mid-Coast, but collectively, the wetlands in the area are important[8]. Shorebirds were most abundant at Brazoria National Wildlife Refuge (BNWR), San Bernard National Wildlife Refuge (SBNWR), and Matagorda Island National Wildlife Refuge (MINWR) (Fig. 5). Maximum counts at BNWR and SBNWR exceeded 45,000 with a maximum count of nearly 26,000 at MINWR. Piping Plover were also abundant at MINWR with a maximum count January–June of 303.

Some of the largest expanses of undisturbed wetland complexes in the Western Hemisphere are found in the Laguna Madre of Texas and Mexico[19] and this area is one of the most significant areas for aquatic-bird life on the Gulf of Mexico Coast [1,10]. Areas where shorebirds concentrate include Laguna Atascosa (LANWR) and Lower Rio Grande Valley National Wildlife Refuge, beaches and washover passes of Padre Island and Mustang Island, the vast algal and non-algal tidal flats in Laguna Madre and South Bay (SB)[8]. More than 100,000 shorebirds were counted during a single survey of LANWR (Fig. 5)[6]. Although a maximum count of nearly 20,000 birds was counted on Padre Island National Seashore (PINS) beaches and tidal flats during April 1998[8], it is likely that the maximum count is much greater during winter and early spring when these flats are used most heavily[20]. The majority of the threatened Great Plains population of Piping Plover probably winter in Laguna Madre[13] using both barrier islands and tidal flats[14,15,16,17,20]. Maximum counts of Piping Plover from 65–200 were obtained during surveys of the barrier island beaches and tidal flats in the Laguna Madre region[6].

## Habitat Use in Texas

Five general coastal habitats[21,22,23,24,25,26] are likely to be used by shorebirds in Texas[8]. The beach habitat contains the marine intertidal or “swash” zone of the barrier islands characterized by sand substrate, strong-wave action, regular tidal fluctuations, and marine salinity (35 ppt), as well as berm habitats above mean sea level (MSL). This habitat is used by 15 species (Table 5), notably the plovers. The intertidal habitat is used primarily for foraging and the areas above MSL primarily for roosting[27]. Texas barrier islands do not appear to be used as nesting habitats for any shorebird species. On Padre Island National Seashore, 30 shorebird species have been documented[27,28]. The most common and abundant shorebird was Sanderling. Total abundance generally peaks during fall migration (August–September) with a smaller peak during spring (March–April). Few shorebirds are present in early winter (December–January) or June.

Washover passes on the barrier islands are used as nesting habitats for a few shorebirds such as Snowy Plover and are important roost sites for plovers and other species[16,29,30,31]. These non-vegetated, channel-like landforms cut through barrier islands into the lagoon, terminating in fan-shaped sand and mud tidal flats. They are caused when the tides and winds from strong storms (usually tropical systems) breach the island and cause a temporary pass between the Gulf and bays that usually fill in within a few weeks or months after the storm.

**TABLE 5**
**Shorebird Coastal and Estuarine Habitat Use in Texas[8,43,45]**

| Species | Beach | Washover | Tidal Flat | Reef Flank | Marsh |
|---|---|---|---|---|---|
| Black-bellied Plover | X | X | X | | X |
| American Golden-Plover | X | X | | | X |
| Snowy Plover | X | X | X | | X |
| Wilson’s Plover | X | X | X | | X |
| Semipalmated Plover | | X | X | | X |
| Piping Plover | X | X | X | | |
| Killdeer | | | X | | X |
| American Oystercatcher | X | | X | X | |
| Black-necked Stilt | | | X | | X |
| American Avocet | X | | X | | X |
| Greater Yellowlegs | X | X | X | | X |
| Lesser Yellowlegs | | X | X | | X |
| Solitary Sandpiper | | | X | | |
| Willet | X | X | X | X | X |
| Whimbrel | | | X | | X |
| Long-billed Curlew | X | | X | | X |
| Hudsonian Godwit | | | X | | X |
| Marbled Godwit | X | | X | | |
| Ruddy Turnstone | X | X | X | X | |
| Red Knot | X | X | X | | X |
| Sanderling | X | X | X | | |
| Semipalmated Sandpiper | | | X | | X |
| Western Sandpiper | X | X | X | X | X |
| Least Sandpiper | | X | X | | X |
| White-rumped Sandpiper | | | X | | |
| Baird’s Sandpiper | | | X | | |
| Dunlin | X | X | X | | X |
| Stilt Sandpiper | | | X | | |
| Dowitcher | | | X | | X |
| Wilson’s Phalarope | | | X | | X |

Although they are only rarely flooded, wind erosion causes the “channel” to persist. Fifteen species, and all plovers except Killdeer, use this habitat (Table 5). It probably functions mostly as a roosting site or high-tide refuge for most species. There is little information concerning abundance or seasonal use of this habitat.

Tidal flats include vast, gradually sloping bayside habitats from about 1 m below MSL to 2 m above MSL, and smaller fringing areas bordering bay margins and tidal creeks. The largest expanses of tidal flats are generally located on the bay sides of barrier islands, with smaller tidal flats on mainland bay shores. Salinities are estuarine (20–25 ppt) to hypersaline (>40 ppt) and substrates range from sand to mud. Timing and persistence of tidal flooding and exposure is unpredictable because it is governed by winds; astronomical tidal fluctuations are generally negligible. Vegetation is generally limited to blue-green algal mats of varying thickness growing on the surface of the substrate. Thirty-seven shorebird species have been documented on tidal flats on the central Texas Gulf coast[32]. All shorebirds except American Golden Plover use tidal flats (Table 5), and the habitat is a particularly important winter foraging habitat for Piping and Snowy plovers [14,15,16,17,20].

Shorebirds can be extremely abundant on exposed tidal flats along the central and southern Texas coast during winter and migratory periods and use the habitat primarily as foraging habitat[1,13,20,33,34,35,36,37,38,39,40]. A few shorebirds nest on the flats, notably Snowy[31] and Wilson’s plovers. Shorebirds were abundant on mainland tidal flats from August to April or May[33,35,41] and on barrier-island tidal flats adjacent to Laguna Madre from November to March or April[20; 34,37,38,39,40,42]. In general, peak numbers during fall migration were lower than peak wintering or spring migration numbers, probably due to decreased exposed area caused by fall high tides[20]. Overall, sandpipers and plovers are the most common and abundant species.

The reef flank is an estuarine habitat that is exposed during low tides on the edges and between live and dead oyster reefs[8]. The substrate is muddy, but contains broken shells and some living and dead oyster clumps. Salinity ranges from 10–30 ppt. Only four species use this habitat (Table 5). Seasonal abundance of shorebirds in this habitat has not been studied.

The marsh habitat is found in the intertidal area as a generally narrow fringe of vegetation on the margins of bays and estuaries, primarily north of the Colorado River. Substrates are primarily muddy and vegetated, most often with smooth cordgrass (*Spartina alterniflora*), glasswort (*Salicornia* spp.), and occasionally other cordgrass species (*Spartina* spp.). Vegetation may be persistent (usually cordgrass) or non-persistent (other species). Twenty-one species use the marsh habitat, mostly larger, longer-legged species such as Black-Necked Stilt and some sandpipers (Table 5) and many of the smaller birds, such as the sandpipers and plovers, may be confined to patches of non-vegetated flat habitat within the marsh mosaic. Seasonal abundance within these habitats is not well known, but in general, numbers are greatest during spring and fall migration and lowest during summer[41,43].

For Piping Plover, the unique juxtaposition of beach, washover, and tidal flat habitat along the Texas Coast and in some other areas of the Gulf Coast (e.g., Alabama) appears critical to wintering populations[5,14,15,17,44]. Piping Plover regularly moved between habitats, largely in response to flooding and exposure of barrier island tidal flats, with numbers decreasing on flats as they were inundated by tides[14,17,39]. Beaches were preferred secondary habitat on the upper and middle coast, but mainland flats and washover passes were preferred over beaches in lower Laguna Madre. Barrier island beaches, mainland flats, and washover passes in lower Laguna Madre were critical high-tide refuges and roosting areas.

Wetland use by shorebirds along the Texas coast was delineated[45] using wetland subclasses designated by Cowardin et al.[46]. Estuarine wetlands were used by 29 shorebird species (Table 6), although most species also used freshwater wetlands to some extent. Of the wetland types used, all but Common Snipe, Upland Sandpiper, and Killdeer used at least 50 percent estuarine wetland types, and estuarine wetlands were among the top five wetlands in both density and proportion of birds foraging for all species for which information was provided. In general, intertidal wetlands (abbreviations beginning with E2) were most often among the top five in both density and proportion of birds foraging. Subtidal (abbreviations beginning with E1) were commonly used by larger, longer-legged species such as American Avocet and Willet. Eleven species exhibited preferences for or avoided some habitats. The habitat frequently preferred was estuarine intertidal unconsolidated shore sand or mud (E2US3 or E2US2), and the habitats frequently avoided were estuarine subtidal aquatic bed rooted vascular (E1AB3) and estuarine intertidal emergent persistent (E2EM1).

Zone or microhabitat use by shorebirds in southern Texas barrier-island and tidal-flat habitats has been studied by several authors[20,27,28,34,35,38]. Zones are typically defined as bands of conditions running parallel to the water's edge and determined by tide stage and the degree to which sediments are wetted or covered with water, or the depth of the water in relation to a shorebird's leg length[47], e.g., subtidal, intertidal, and supratidal zones on a barrier-island beach. Microhabitats may or may not be analogous to zones, but are typically determined by the degree to which sediments are wetted or covered with water or the depth of the water in relation to a shorebird's leg length. On many tidal flats, due to microrelief within the flat, microhabitats exist as a mosaic flat rather than as distinct bands of conditions running parallel to the water's edge[20,40].

On barrier islands, shorebirds tend to concentrate in the damp foreshore and "swash" zone where they forage for invertebrates[27,28]. Piping and Snowy plovers, Ruddy Turnstone, and a few other species forage along the berm and in the wrack line. The dry backshore area is used rarely, but primarily as resting or roosting habitat.

**TABLE 6**
**Shorebird Estuarine Wetland Use on the Texas Coast**

| Species | Wetland Types | % Estuarine | Top 5 Density[a] | Top 5 Proportion Foraging[a] | Selection/ Avoidance |
|---|---|---|---|---|---|
| Black-bellied Plover | 17 | 88.2 | E2RF2, E2RS2, E2US4, E1RF2, E1UB1 | E2RS2, E2RF2, E2US4, E1RF2, E2US1 | E2US3 +<br>E2EM1 -<br>E1UB3 - |
| American Golden-Plover | 11 | ? | E2US4 | E2US4 | |
| Snowy Plover | 8 | ? | E2US1, E2US2, E2US4 | E2US4, E2US2, ESAB3 | |
| Wilson's Plover | 10 | ? | E1UB4, E2US2, E2US4 | E1UB4, E2US2, E2US4 | |
| Piping Plover | 8 | 100 | ? | ? | |
| Killdeer | 48 | 37.5 | E1UB1, E2US4 | E2US4 | E2EM1 -<br>E1AB3 -<br>E1UB3 - |
| American Oystercatcher | 9 | ? | E2RS2, E2US1, E2RF2 | ? | ? |
| Black-necked Stilt | 27 | 48 | E2AB3, E2US4, E2EM2, E2AB1, E2US3 | E2AB1, E2EM2, E2AB3, E1UB2, E2US3 | E2EM1 -<br>E1AB3 - |
| American Avocet | 20 | 60 | E1UB1, E2EM2, E2US4, E2US3 | E1UB1, E2EM2, E2US4, E2AB3, E2US1 | E2US3 +<br>E1UB3 - |
| Greater Yellowlegs | 27 | 44.4 | E2US4, E2AB3, E1RF2, E2US3 | E2US4, E1RF2, E1AB3, E2US3 | E2AB3 +<br>E2US3 + |
| Lesser Yellowlegs | 28 | 53.6 | E2US4, E1UB1, E2AB1, E2RS2 | E2US4, E1UB1, E2AB1, E2RS2 | E2US3 +<br>E1AB3 - |
| Solitary Sandpiper | 16 | ? | E2RF2, E2US4 | E2RF2, E2US4 | |
| Willet | 23 | 73.9 | E2RS2, E1UB1, E1RF2, E2US1, E2RF2 | E2RS2, E2RF2, E2EM2, E1UB1 | E2US2 +<br>E2AB3 +<br>E2US3 + |
| Upland Sandpiper | 4 | 25 | ? | ? | |
| Whimbrel | 8 | ? | E2US2, E1AB1, E1UB3, E1AB3 | E2US2, E1AB1, E1UB3, E1AB3 | |
| Long-billed Curlew | 23 | 56.5 | E2EM2, E2US4, E1UB2, E1UB4, E2AB3 | E2EM2, E1UB2, E1UB4, E2US4, E2US1 | |
| Marbled Godwit | 8 | 100 | ? | ? | |
| Ruddy Turnstone | 10 | ? | E2RS2, E2US1, E2RF2, E2EM2, E2AB1 | E2RS2, E2US1, E2RF2, E2EM2, E2AB1 | |
| Red Knot | 10 | ? | E2EM2, E2US4 | E2EM2, E2US4, E2US3 | |
| Sanderling | 12 | ? | E2RF2, E2US1, E2RS2, E2US2 | E2RF2, E2US1, E2RS2, E2US2 | |
| Semipalmated Sandpiper | 7 | 85.7 | ? | ? | |
| Western Sandpiper | 34 | 50 | E1RB1, E2US4, E1UB1 | E2US4, E1UB1 | E2US3 +<br>E1AB3 - |
| Least Sandpiper | 20 | 50 | E1UB4, E2RS2, E2AB3, E2US4 | E2RS2, E2AB3, E1UB4, E2US4 | E2AB3 -<br>E2US3 + |
| White-rumped Sandpiper | 7 | ? | E2AB3, E2US3 | E2AB3, E2US3 | |
| Dunlin | 9 | ? | E2RF2, E2AB3, E2US3 | E2AB3, E2US2, ESUS3 | |
| Stilt Sandpiper | 7 | ? | E2UB4, E2UB2, E2AB3 | E2UB4, E2UB2, E2AB3 | |
| Dowitchers | 29 | 51.7 | E1UB1, E2AB3, E2US3 | E1UB1, E2AB3, E2US3 | E2US3 +<br>E2EM1 - |
| Common Snipe | 25 | 36 | E2SS1, E2AB3 | E2SS1, E2SB3 | E1UB3 - |

Summarized from [45].
Habitat abbreviations are wetland subclasses [46]. Abbreviations for estuarine subtidal habitats begin with E1 and are further classified using the following letter/number combinations: RB1 = rock bottom bedrock; UB1 = unconsolidated bottom cobble-gravel; UB2 = unconsolidated bottom sand; UB3 = unconsolidated bottom mud; UB4 = unconsolidated bottom organic; AB1 = aquatic-bed algal; AB3 = aquatic-bed rooted vascular; AB4 = aquatic-bed floating vascular; RF2 = reef mollusc. Abbreviations for estuarine intertidal habitats begin with E2 and are further classified using the following letter/number combinations: AB1 = aquatic-bed algal; AB3 = aquatic-bed rooted vascular; RF2 = reef mollusc; SB3 = streambed mud; RS2 = rocky shore rubble; US1 = unconsolidated shore cobble-gravel; US2 = unconsolidated shore sand; US3 = unconsolidated shore mud; US4 = unconsolidated shore organic; EM1 = emergent persistent (vegetation); EM2 = emergent non-persistent (vegetation); SS1 = scrub-shrub broad-leaved deciduous (vegetation). ? = no information provided.

The majority of shorebirds on tidal flats in the Laguna Madre can be grouped into a shallow-probing, surface-feeding guild, whose members are primarily visual foragers. This guild can be further divided into three microhabitat guilds based on apparent microhabitat preferences[20]. The three microhabitats exist as a mosaic within these flats and are characterized as follows: open water — areas covered by at least 5 cm of water; intertidal — areas at the water's edge in less than 2–4 cm of water; wet — areas with wet or saturated sediments and less than 2 cm of standing water; damp — areas that appear damp or dry on the surface but with wet sediments below the surface. Sandpipers and Black-Bellied Plover dominated the guild that preferred the wet microhabitat. Piping, Snowy, and Semipalmated plovers preferred the wet and damp microhabitats in nearly equal proportions. Longer-legged, longer-billed shorebirds such as Dunlin, Willet, and Dowitchers preferred open water microhabitats. Only Western Sandpipers exhibited a preference for the intertidal microhabitat. Similar microhabitats and species preferences were found in studies of tidal flats in lower Laguna Madre[38] and Oso Bay[35].

## NORTHEASTERN GULF

Thirty species of shorebirds have been reported in the Northeastern Gulf region (Table 3) with the area ranked as an "area of extremely high importance relative to other regions" for 15 species and "of considerable importance" to 12 others[48]. Four species are considered "extremely high priority": Snowy Plover, Piping Plover, American Oystercatcher, and Red Knot. The wintering range of American Oystercatcher is restricted to the Atlantic and Gulf coasts of Florida (Table 2).

Much of the northern Gulf Coast from Mississippi to Florida Keys consists of primarily of low to medium energy vegetated shorelines with salt marshes in temperate areas of the northern Gulf to just north of Tampa Bay, Florida, and mangroves and everglades in subtropical to tropical Peninsular Florida[9,49]. The occurrence of bays and inlets and sandy beaches varies. Four distinct regions have been characterized in this area (Fig. 6)[9]. From the Louisiana-Mississippi border to the Appalachicola River (Panhandle), the coast is characterized by medium-energy habitats, including narrow barrier island or peninsular beaches and small bays and inlets fringed by estuarine marshes or tidal flats. Salt marshes are characterized by smooth cordgrass between

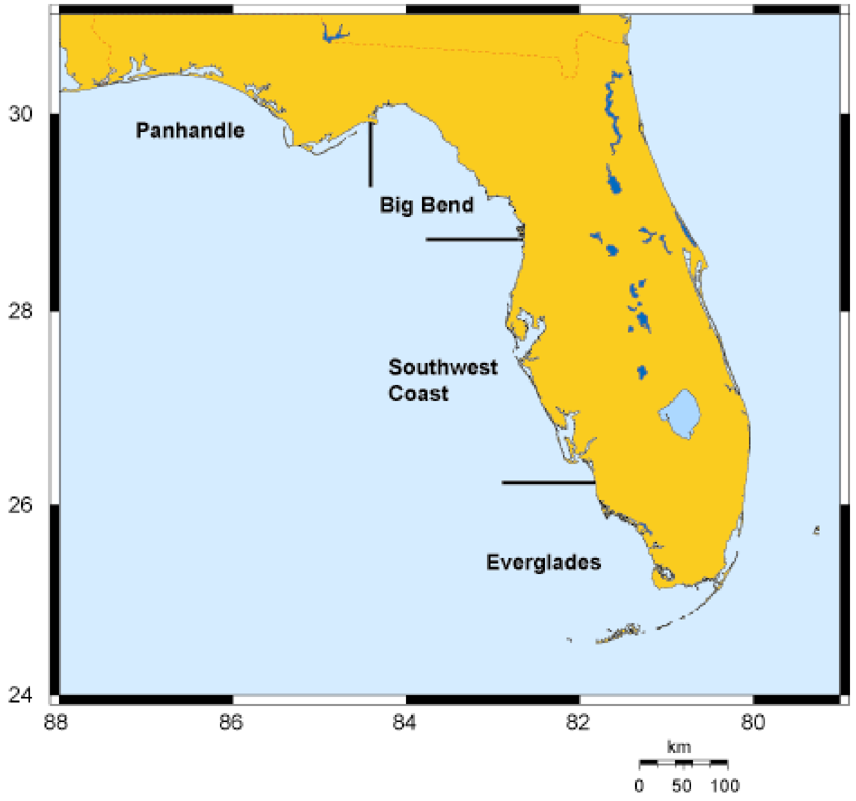

**FIGURE 6.** Regional habitat types within the Northeastern Gulf of Mexico[9].

the intertidal and mean high water (MHW) and black needle rush (*Juncus roemerianus*) above MHW[49]. The Big Bend region of Florida is characterized by low energy and extensive stands of cordgrass marsh. Bays and inlets are uncommon, and there are no barrier islands or sandy beaches. The Southwest region includes the Tampa Bay-Orlando metropolitan area and is extremely developed along the coast. The habitats in this area are similar to those in the Panhandle. Bays and inlets are common with medium-energy sandy beaches associated with islands and peninsulas. The Everglades region is similar to the Big Bend, except the tropical counterpart of the salt marsh, mangroves, primarily red mangrove (*Rhizophora mangle*) and black mangrove (*Avicennia germinans*), replace the salt marsh vegetation.

There are no published accounts of shorebird abundance or distribution for this region outside Florida, and even within Florida, the majority of studies have been limited to only one or a few sites[50,51,52] or one species[44,53,54]. Identification and conservation of wintering sites in Florida are a conservation priority due to the rapid development of coastal property, so a comprehensive survey of wintering shorebird abundance and habitat use was undertaken in winter 1993–94[9]. Twenty-five species were documented during the survey with a total of 27,900 counted along the Gulf Coast. In each region, there was at least one site with a maximum mean (mean + 1 SD) of at least 3,000 individuals (Fig. 7). Shorebirds were most abundant at Lanark Reef (LR) in the Panhandle, Cedar Key (CK) in the Big Bend region, Anclote Key (AK) in the Southwest Coast region, and Lake Ingraham (LI) in the Everglades. Maximum means were highest overall in two sites in the Everglades, Lake Ingraham and northwest of Palm Key (NPK). Despite the high degree of development, shorebirds were abundant in the Southwest Coast, particularly around Tampa Bay, and there were more sites with suitable shorebird habitat[25] than in the other regions[6,7,8,9,10,11,12,13,14]. In general, individual species abundance was also greatest in the Southwest region and least in the Big Bend Region (Table 7). Species richness was greatest in the Panhandle region and least in Big Bend and Everglades regions. Statewide (including the northeastern Atlantic Coast), a significant proportion of the U.S. populations of Willet, Marbled Godwit, Dunlin, American Oystercatcher, Snowy Plover, Wilson's Plover, and Piping Plover winter in the state.

## MEXICO

The Gulf Coast of Mexico can be divided into four regions (Fig. 8): Laguna Madre, Tampico/Veracruz, Alvarado/Terminos, and Yucatan. Habitats within the Laguna Madre de Tamaulipas are similar to those in the Laguna Madre of Texas with large expanses of unvegetated tidal flats and well-developed barrier islands. Near Rio Soto La Marina, small amounts of red mangrove are also found. South of Laguna Madre de Tamaulipas (Tampico/Veracruz and Alvarado/Terminos), most coastal lagoons seemed to have little suitable shorebird habitat[10]. The lagoons of these regions are fluvial-deltaic in origin, with generally poorly developed barrier islands (Cabo Rojo is the exception), but some open Gulf beach habitat. The lagoons are relatively small and vegetated with mangroves, trees or other vegetation down to the water's edge with little exposed mud available. The Yucatan region is characterized by extensive coastal wetlands with large areas of non-vegetated habitats contained within the wetland mosaic. There are some barrier-island and open Gulf-beach habitats along the Yucatan Gulf Coast.

During aerial surveys of the Mexican Gulf Coast, 24 shorebird species were encountered[10]. Shorebird numbers were highest in Laguna Madre de Tamaulipas (LM; 83,338), followed by Tampico (TP) lagoons (9,407), Campeche (CA) wetlands (6,732), and the Puerto Progresso (PP) wetlands and coastline (3,835) (Fig. 9). Laguna Madre de Tamaulipas held nearly 68 percent of the shorebirds counted on the Mexican Gulf Coast with the area north of Laguna Alvarado constituting 80 percent of the total. Few shorebirds were found wintering in the

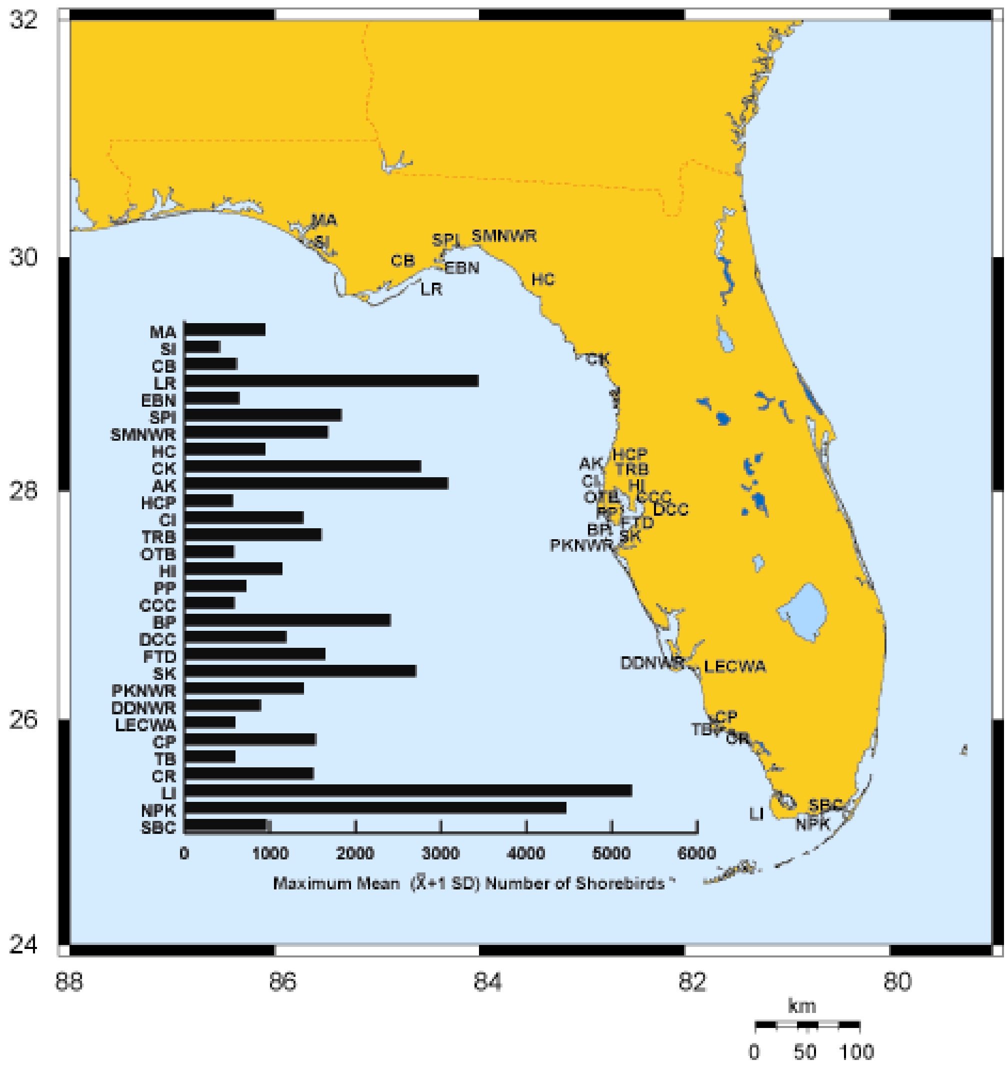

**FIGURE 7.** Shorebird abundance and distribution in Florida presented as the maximum mean (mean + 1 SD) of individuals counted at each site between December 1993 and March 1994[9]. Only sites with a maximum mean ≥ 500 are shown. MA = Marifarms; SI = Shell Island; CB = Carabelle Beach; LR = Lanark Reef; EBN = east of Bay North; SPI = Sprague Island; SMNWR = St. Marks National Wildlife Refuge; HC = Hagens Cove; CK = Cedar Key; AK = Anclote Key; HCP = Howard County Park; CI = Caledesi Island; TRB = Three Rooker Bar; OTB = Old Tampa Bay; HI = Honeymoon Island; PP = Point Pinellas; DCC = Delany Creek Canal; FTD = Fort Desoto; SK = Shell Key; PKNWR = Passage Key National Wildlife Refuge; DDNWR = Ding Darling National Wildlife Refuge; LECWA = Little Estero Coastal Wildlife Area; CP = Capri Pass; TB = Tigertail Beach; CR = Cape Romano; LI = Lake Ingraham; NPK = Northwest of Palm Key; SBC = Snake Bight Channel.

extensive coastal wetlands of the northern Yucatan Peninsula. Overall, sandpipers (*Calidris* spp.) were the most abundant species. Firm, non-vegetated substrate was the habitat most often used by shorebirds wintering in Mexico (Table 8). Sanderlings were found exclusively on beaches, and Ruddy Turnstones on either sandy or rocky shores, but the other species that used beaches (Black-Bellied Plover, American Oystercatcher, Willet) also used wetland habitats.

## Laguna Madre de Tamaulipas

The distribution and abundance of shorebirds in Laguna Madre de Tamaulipas is not well known, however, it is likely that the tidal flats and barrier island beaches are used similarly to those in Texas. Twenty-four species have been documented within the lagoon[10,55,57]. This area

**TABLE 7**
**Species Distribution and Relative Abundance in Gulf Coast Regions of Florida Arranged from Most Abundant to Least Statewide**

| Species | Panhandle | Big Bend | Southwest | Everglades | % National Total |
|---|---|---|---|---|---|
| Dunlin | 2 | 3 | 1 | 4 | 8.1 |
| Western Sandpiper | 3 | 4 | 2 | 1 | 4.6 |
| Sanderling | 2 | 4 | 1 | 3 | 3.4 |
| Short-billed Dowitcher | 4 | 2 | 1 | 3 | 3.5 |
| Willet | 2 | 4 | 1 | 3 | 18.3 |
| Red Knot | 3 | | 1 | 2 | 1.2 |
| Black-bellied Plover | 2 | 4 | 1 | 3 | 2.5 |
| Semipalmated Plover | 3 | 4 | 1 | 2 | 2.5 |
| Ruddy Turnstone | 2 | 4 | 1 | 3 | 0.5 |
| Marbled Godwit | 1 | 4 | 2 | 3 | 22.8 |
| Wilson's Plover | 3 | | 1 | 2 | 29.4 |
| Least Sandpiper | 3 | 4 | 2 | 1 | 0.1 |
| Piping Plover | 2 | 4 | 1 | 3 | 13.3 |
| American Oystercatcher | 1 | 3 | 2 | | 8.1 |
| Snowy Plover | 2 | | 1 | 3 | 5.9 |
| Lesser Yellowlegs | | 2 | 3 | 1 | 0.1 |
| Killdeer | 3 | 2 | 1 | | 0.2 |
| American Avocet | | | 1 | | 0.3 |
| Greater Yellowlegs | 2 | 1 | 3 | | 0.3 |
| Whimbrel | 3 | 2* | 1 | 2 * | 0.6 |
| Black-necked Stilt | | | 1 | | |
| Spotted Sandpiper | | | 1 | | |
| Long-billed Curlew | | | 1 | | |
| Common Snipe | | 1 | | | 0.1 |

*Tie.

Data from [9].

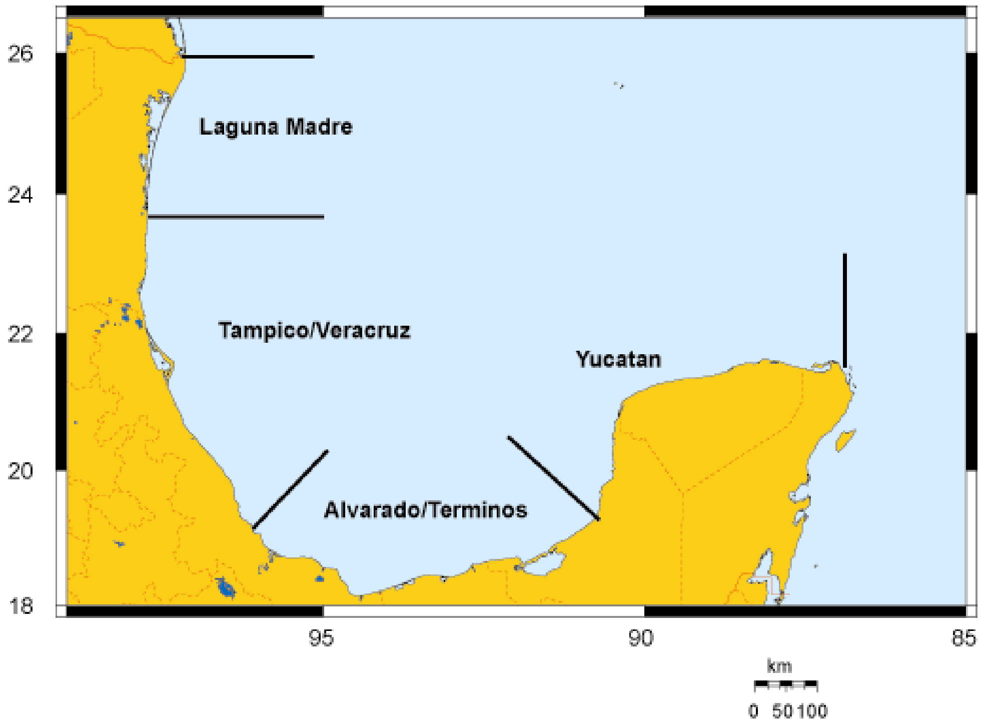

**FIGURE 8.** Regional habitat types along the Mexico Gulf Coast.

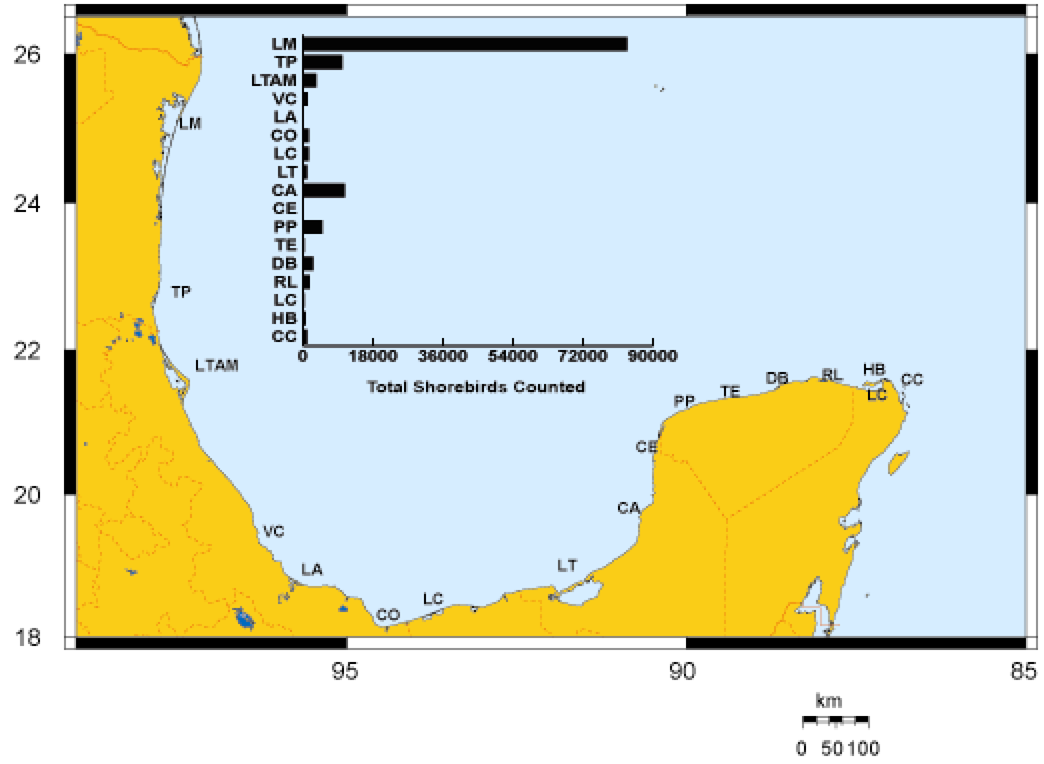

**FIGURE 9.** Shorebird abundance and distribution along the Mexico Gulf Coast presented as the total individuals counted during a survey in January 1993[10]. LM = Laguna Madre de Tamaulipas; TP = Tampico lagoons and shoreline; LTAM = Laguna Tamiahua lagoon and shorelines; VC = lagoons and shorelines from Laguna Tamiahua to Veracruz; LA = Laguna Alvarado and Alvarado coastline; CO = Coatzacoalcos coast; LC = Laguna Carmen and coastlines to Laguna de Terminos; LT = Laguna de Terminos; CA = wetlands and coastlines from Laguna de Terminos to Punta Nimun, Campeche; CE = Celestun estuary and coast; PP = wetlands and coastlines between Celestun and Puerto Progresso; TE =wetlands and coastlines from Puerto Progresso to Telchac Puerto; DB = wetlands and coastlines from Telchac Puerto to Dzilam de Bravo including San Felipe; RL = wetlands and coastline of Rio Lagartos; LC = Laguna Conil; HB = wetlands and coastlines from Holbox to Cabo Catoche; CC = wetlands and coastlines from Cabo Catoche to Cancun.

contained the only substantial numbers of sandpipers and banded plovers on the Mexican coast and was also used extensively by dowitchers. Most shorebirds were found on the extensive mainland and barrier-island tidal flats[10]. Barrier-island beaches were also used by shorebirds with both wintering and nesting birds reported. Sanderlings were the most abundant winter resident. Snowy and Wilson’s plovers were found to nest in the broad coppice dune zone on beaches between the Rio Grande and Mezquital, Tamaulipas[29].

The coast of Tamaulipas north of the Rio Soto la Marina has been divided into three zones[55]: northern, consisting of isolated mudflats and ephemeral wetlands between in the Rio Grande and the northern shoreline of Laguna Madre de Tamaulipas that has been described as a broad tidal flat that is primarily inundated in response to rainfall[56]; central, consisting of the tidal flats and barrier island habitat of northern Laguna Madre; and southern, consisting of the tidal flats and barrier island habitat of the southern Laguna Madre. Black-Bellied Plover, Willet, Sanderling, Western Sandpiper, and Least Sandpiper were found throughout the area and in all zones. Snowy and Wilson’s plovers were found only in the southern zone. Shorebirds were more common in the southern zone during fall migrations than they were during winter[57]. Of the 18 species documented, 11 were described as year-round residents. Six wintered or were found only during spring migration. Wilson’s Plovers were found only during the summer and breed in the area.

**TABLE 8**
**Abundance, Distribution and Habitat Use of Selected Species on the Mexican Gulf Coast[10]**

| Species | Most Abundant | Habitat |
|---|---|---|
| Sandpipers | 93% in top 5 sites (see text) | unvegetated areas within wetland complexes |
| Sanderlings | Laguna de Terminos to Champoton | beaches |
| Black-bellied Plover | Laguna Madre | beaches; firm muddy areas of wetlands & lagoons |
| Ruddy Turnstone | Laguna de Terminos to Champoton | rocky or sandy shore with wrack |
| Yellowlegs | western & northern Yucatan coastline | lagoons & wetlands behind outer coast |
| Dowitchers | Laguna Madre | lagoons & wetlands |
| American Oystercatcher | Laguna Madre | beaches; firm muddy substrates in wetlands |
| Black-necked Stilt | northern Yucatan and Rio Lagartos | brackish & freshwater wetlands |
| American Avocet | Laguna Madre | open pans in wetlands; brackish coastal habitats |
| Marbled Godwit | Laguna Madre | mudflats |
| Whimbrel | Laguna Madre & Laguna de Terminos | ? |
| Long-billed Curlew | Laguna Madre & Laguna Tamiahua | ? |
| Willet | Laguna Madre | beaches; firm substrates in wetlands |

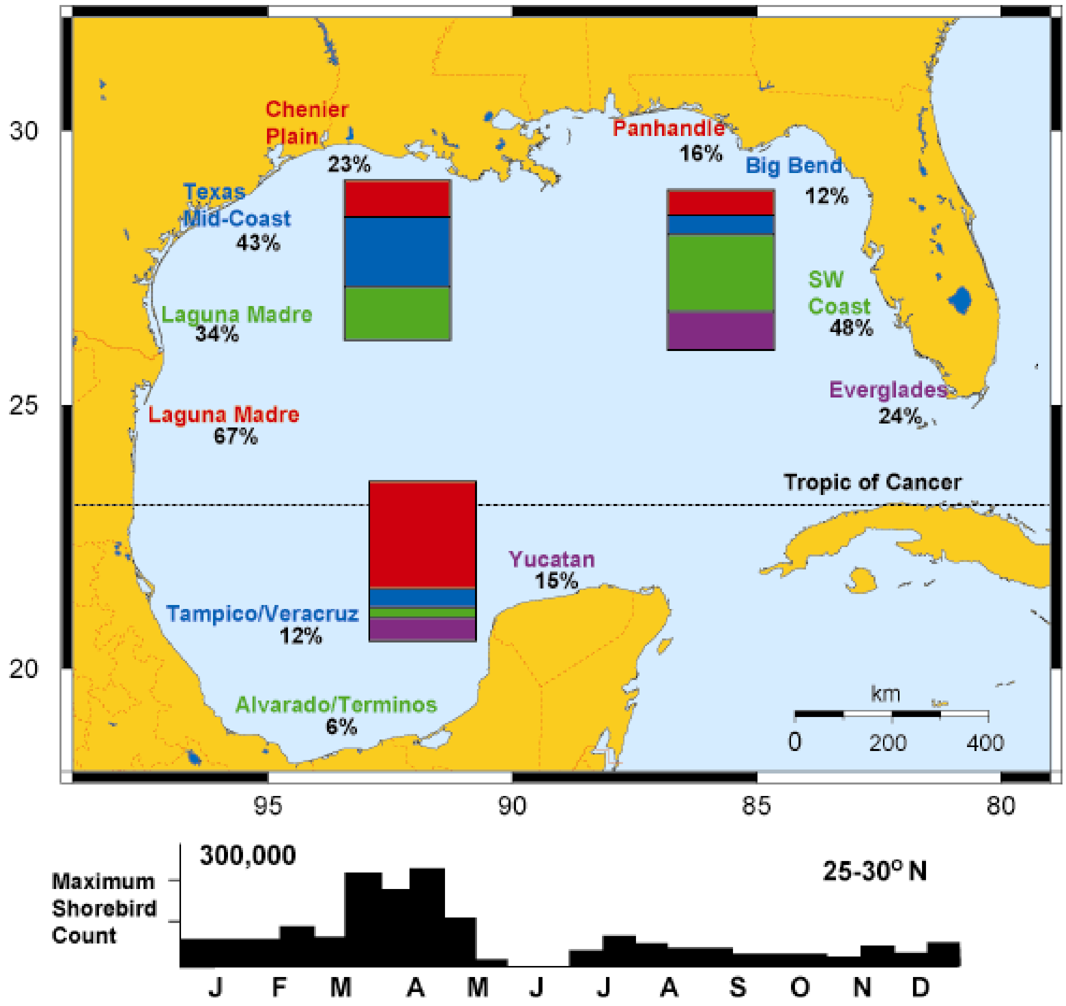

**FIGURE 10.** Comparison of relative abundances of shorebirds by habitat in the three regions of the Gulf of Mexico using data from [8,9,10] with maximum monthly counts in the 5° latitudinal band from 25-30° N[6].

## REGIONAL OVERVIEW AND COMPARISONS

Since data were not collected in comparable ways throughout the region, direct comparisons of abundances among regions cannot be made. The maximum-mean shorebird abundances presented for Florida are generally less than one-fifth the maximum counts presented for the Northwestern Gulf, so it seems likely that overall abundances in the Northeastern Gulf are much less than in the Northwestern Gulf. Similarly, the total shorebird counts in Mexico south of Laguna Madre de Tamaulipas are much less than the maximum counts in the Northwestern Gulf, so overall abundances are likely to be much less as well. The general pattern of relative abundance is to increase from north to south (Fig. 10), then decrease south of the Tropic of Cancer (23º 27' N). Overall, shorebirds in the Gulf of Mexico are most abundant between ~23º N and ~ 29º N (west: Texas Mid-Coast to Tampico Lagoons; east: Florida Southwest Coast-Everglades). Based on bimonthly maximum counts within 5º latitudinal ranges[6], the region between 25 and 30º N is used most heavily by wintering and spring migrating birds.

## CONCLUSION

- Wintering and migrating shorebirds are most abundant along the Gulf Coast in Texas and Tamaulipas, particularly the Laguna Madre ecosystem. Other important areas are the Southwest Coast region of Florida and the Yucatan region of Mexico.
- In general, relative abundances of shorebirds increase from north to south, and decrease south of the Tropic of Cancer (23º 27' N). Based on bimonthly maximum counts within 5º latitudinal bands, the region between 25 and 30º N is used most heavily by wintering and spring migrating birds.
- Non-vegetated coastal wetland habitats associated with bays, inlets and lagoons, particularly tidal flats, and sandy beaches are the habitats that appear to be favored by wintering and migrating shorebirds. In general, these habitats tend to occur as habitat complexes that allow for movement between them in relation to tidal flooding of bayshore habitats. This relationship is particularly important to Piping Plover and may be important to others.
- Although vegetated habitats are used by some species, they do not appear to attract large numbers of birds. This habitat is most widespread between the Texas-Louisiana border and the Florida Panhandle region, but it has not been studied extensively. Shorebird abundance and habitat use in this area need to be addressed.

## ACKNOWLEDGEMENTS

The comments of an anonymous reviewer and Tim Brush improved the manuscript greatly. This is Texas A&M University-Corpus Christi, Center for Coastal Studies Contribution No. 54.

## BIOSKETCH

**Kim Withers**, Ph.D., is a Research Scientist at the Center for Coastal Studies, Texas A&M University - Corpus Christi and an Adjunct Professor of Biology and Environmental Science, Texas A&M University. She received her Ph.D. from the Department of Wildlife Fisheries Sciences, Texas A&M University in 1994. Her dissertation was titled, “Relationship of Macrobenthic Prey Availability to Shorebird Use of Blue-Green Algal Flats in the Upper Laguna Madre (Texas)”. Dr. Withers has studied shorebird ecology on the middle Texas Gulf of Mexico coast for the last 15 years. Her research interests include tidal flat ecology, particularly relationships between shorebirds and invertebrate prey resources; seagrass ecology and invertebrate communities; impacts of seagrass succession on juvenile fish communities; mangrove-associated faunas including juvenile fish; fauna of Caribbean ironshores; benthic ecology; fisheries resources in Texas and Mexico; and biofouling communities of offshore oil platforms.
